# A Current Approach to Logistic Regression Analysis of Birth Order and Sexual Orientation

**DOI:** 10.1007/s10508-025-03275-3

**Published:** 2025-12-09

**Authors:** Ray Blanchard, Jan Kabátek, Bozena Zdaniuk

**Affiliations:** 1 Department of Psychiatry, University of Toronto, Toronto, ON Canada; 2 Melbourne Institute of Applied Economic and Social Research, University of Melbourne, Melbourne, VIC 3053 Australia; 3 ARC Centre of Excellence for Children and Families over the Life Course, Institute for Social Science Research, University of Queensland, Brisbane, Australia; 4 Institute of Labor Economics, Bonn, Germany; 5 CentER, Tilburg University, Tilburg, The Netherlands; 6 Department of Obstetrics and Gynaecology, University of British Columbia, Vancouver, BC Canada

**Keywords:** Birth order, Family size, Fertility, Homosexuality, Logistic regression, Sexual orientation

## Abstract

Numerous statistical procedures have been developed to examine the statistical relations between quantifiable aspects of an individual’s sibship and the likelihood of that individual manifesting a homosexual preference. Our purpose in this methodological paper is explaining how to use and how to interpret the multiple regression approach introduced by Ablaza et al. (2022), modified by Blanchard (2022), and reorganized by Zdaniuk et al. (2025)—hereafter, the ABZ model. First, we list the sibship variables of present interest (e.g., number of older brothers), summarize their previously observed associations with sexual orientation, and discuss the language and labels that we recommend for describing empirical results in this research area. We then explain, in concrete, practical terms, how to analyze these sibship variables using the ABZ method, and we present a model analysis using previously published data. Our subsequent sections, which go more deeply into the topic, include a discussion of the mathematical–statistical rationale behind the Ablaza et al. approach—more specifically, its foundation on the *ceteris paribus* condition of multiple regression in conjunction with a “subsetting” property of the relevant sibship data. Finally, we compare the performance of the ABZ model with an older, more frequently used logistic regression model, and we discuss the potential application of the ABZ model to outcomes other than homosexuality.

## Introduction

One of the oldest areas of research on sexual orientation concerns the statistical relations between quantifiable aspects of an individual’s sibship and the likelihood of that individual manifesting a homosexual preference (Lang, 1936; Slater, 1962). Numerous statistical procedures have been developed to examine these relations. Some of these procedures focus on only one sibship parameter (e.g., the proportions of male and female siblings), and some simultaneously analyze more than one parameter (e.g., the total number of siblings and the subject’s birth order among them). Some procedures have been used in only one or a few studies (Blanchard & Bogaert, 1998), some have been proposed but never used (Blanchard, 2014), and some have been used in multiple studies (Blanchard & Bogaert, 1996b). Some are fundamentally different from each other, whereas others are simply variants of the same underlying approach. All of these methods, however, can be used with any dataset that includes the following five variables: the subject’s sexual orientation, and the subjects’ numbers of older brothers, older sisters, younger brothers, and younger sisters.

The main purpose of this technical note is not to review all the statistical procedures that have been used in this research area but rather to provide a practice-oriented description of the most recently introduced—and, in our opinion, the best currently available—procedure. This is the multiple regression approach introduced by Ablaza et al. (2022), modified by Blanchard (2022), and reorganized by Zdaniuk et al. (2025). The description of the procedure itself will be presented in a later section of this paper, following a more detailed introduction to the variables of present interest. In line with our main purpose, we will be referring to other methods that are closely related to this procedure; however, more analytically distinct approaches (e.g., Raymond et al. 2023), will not be addressed.

A secondary purpose of our manuscript is to explain the rationale behind the interpretation of the ABZ parameter coefficients in greater depth than Ablaza et al. (2022, pp. 674–675) explained it.

A tertiary purpose of this paper is to point out the differences between the aforementioned procedural variants, especially the differences between the original version by Ablaza et al. and the two later versions. We hope this will assist readers in studying the published investigations that have used this general approach so far and in understanding the mostly minor differences between them.

### Sibship Parameters and Their Relation to Sexual Orientation

As noted above, various parameters of sibship composition have been studied in relation to sexual orientation. The present paper is concerned with four of them. The first of these is the subject’s number of older brothers. Numerous studies have found that older brothers are associated with an increased likelihood of homosexuality in later-born subjects (e.g., Blanchard & Skorska, 2022; Gómez Jiménez et al., 2020; Raymond et al., 2023). This phenomenon has been called the fraternal birth order effect. The second parameter is the subject’s number of older sisters. Some—but not all—studies have found that older sisters are also associated with an increased likelihood of homosexuality (e.g., Kangassalo et al., 2011; Schwartz et al., 2010; Semenyna et al., 2023; cf. Blanchard & Bogaert, 1996a, 1996b; Blanchard et al., 1998; Ellis & Blanchard, 2001). This phenomenon has been called the sororal birth order effect. The third parameter is the difference in magnitude between the fraternal birth order effect and the sororal birth order effect.

The fourth parameter is the subject’s total number of siblings—in other words, the subject’s sibship size, excluding the subject him- or herself. A number of studies have examined the relation between a subject’s sibship size and that subject’s odds of homosexuality (e.g., Blanchard, 2021; Blanchard et al., 2020; Iemmola & Camperio Ciani, 2009). Significant positive associations, when observed, have sometimes been called the female fertility effect or female fecundity effect. It is probably best to avoid using such labels, because they presuppose that the observed association has something to do with properties of homosexual subjects’ mothers. Such properties could include a greater physiological capacity to conceive or to carry a fetus to term, or personality factors related to a greater desire for offspring. In fact, between-groups differences in mean sibship size could simply be sampling artifacts related to differences in average family size in the populations from which the homosexual and heterosexual groups were recruited. It is therefore preferable to use a theory-neutral label such as sibship size effect for observed findings and to discuss theoretical explanations separately.

### More on Labels

The concepts of fraternal birth order effect and sororal birth order effect may be thought of as something akin to Platonic essences, that is, concepts that are absolute and unchangeable and that exist independently of real-world measurements. In fact, different procedures for quantifying these effects may lead to subtle differences in the thing being quantified. This is most easily explained by example. Both Blanchard and Bogaert (1996b) and Ablaza et al. (2022) used multiple logistic regression to estimate the magnitude of the sororal birth order effect. In both studies, a dichotomous variable representing the subject’s sexual orientation was the criterion variable, and the subject’s number of older sisters (siblings) along with several other variables were the predictor variables. These “other variables,” which can be regarded for the moment as control variables, were somewhat different in the two studies.

As Ablaza et al. pointed out, the sororal birth order effect computed in the Blanchard and Bogart study could be interpreted (or conceptualized) as the change in the odds of homosexuality produced by adding one older sister to an existing sibship. In contrast, the sororal birth order effect computed in the Ablaza et al. study could be interpreted/conceptualized as the change in odds produced by adding one older sister while simultaneously removing one younger sister (thus keeping total sibship size the same).

In order to facilitate communication among researchers, Kabátek and Blanchard (2025) reserved the terms fraternal birth order effect and sororal birth order effect for discussions at the most general level. For the regression coefficients included in their variant of the Ablaza et al. model, the authors devised a different set of labels. These labels (presented below) were intended to reflect their operational definitions of the corresponding effects. Kabátek and Blanchard’s terminology was adopted by Zdaniuk et al. and will also be the terminology used in the present article.

## Description of the Logistic Regression Approach

The analytical approaches discussed in this article are based on a binary logistic regression model of the following form:

1 $$ P\left[ {Y_{i} = 1|{\mathbf{x}}_{i} ,{{\varvec{\upbeta}}}} \right] = \frac{1}{{1 + \exp ( - {\mathbf{x}}_{i}^{\prime } {{\varvec{\upbeta}}})}} $$

In this equation, *Y* denotes the dichotomous criterion variable that captures the sexual orientation of individual *i*. For the purpose of consistency across studies, the homosexual group should be coded with the higher value (e.g., 1 for homosexual and 0 for heterosexual). The term **x** denotes a set of predictor variables, and ${{\varvec{\upbeta}}}$ denotes the corresponding regression coefficients.

In Blanchard and Bogaert’s (1996b) original model—hereafter, the B&B model—the term ${\mathbf{x}}^{\prime}_{i} {{\varvec{\upbeta}}}$ has the following functional form:

2 $$ \begin{aligned} {\mathbf{x}}_{i}^{\prime } {{\varvec{\upbeta}}} & = \beta_{0} + \beta_{1} N({\text{older}}\;{\text{br}}.)_{i} + \beta_{2} N({\text{older}}\;{\text{sis}}.)_{i} + \beta_{3} N({\text{younger}}\;{\text{br}}.)_{i} \\ & \quad + \beta_{4} N({\text{younger}}\;{\text{sis}}.)_{i} + {\mathbf{z}}_{i}^{\prime } {{\varvec{\upbeta}}}_{5} \\ \end{aligned} $$

Here, the four terms starting with *N* denote counts of older brothers, older sisters, younger brothers, and younger sisters, whereas the term **z** denotes a set of other control variables.

Ablaza et al. (2022) proposed a new logistic regression model approach that deviates from this functional form, in that it simultaneously estimates parameters related to sibship size, sibship sex composition, and birth order. Specifically, the authors replaced the counts of older and younger sisters by the count of total number of siblings, and the count of older siblings, irrespective of their sex:

3 $$ \begin{aligned} {\mathbf{x}}^{\prime}_{i} {{\varvec{\upbeta}}} & = \beta_{0} + \beta_{1} N({\text{sib}}.)_{i} + \beta_{2} N({\text{older}}\,{\text{sib}}.)_{i} + \beta_{3} N({\text{older}}\,{\text{br}}.)_{i} \\ & \quad + \beta_{4} N({\text{younger}}\,{\text{br}}.)_{i} + {\mathbf{z}}^{\prime}_{i} {{\varvec{\upbeta}}}_{5} \\ \end{aligned} $$

Blanchard (2022) slightly modified Ablaza et al.’s original model, following suggestions made by Ablaza et al. themselves. These modifications involved dropping the predictor variable number of younger brothers and adding a second regression equation controlling for the number of older sisters (instead of older brothers) in order to increase the number of estimable parameters:

4a $$ {\mathbf{x}}^{\prime}_{i} {{\varvec{\upbeta}}} = \beta_{0} + \beta_{1} N({\text{sib}}.)_{i} + \beta_{2} N({\text{older}}\,{\text{sib}}.)_{i} + \beta_{3} N({\text{older}}\,{\text{sis}}.)_{i} + {\mathbf{z}}^{\prime}_{i} {{\varvec{\upbeta}}}_{4} $$

4b $$ {\mathbf{x}}^{\prime}_{i} {{\varvec{\upbeta}}} = \beta_{0} + \beta_{1} N({\text{sib}}.)_{i} + \beta_{2} N({\text{older}}\,{\text{sib}}.)_{i} + \beta_{3} N({\text{older}}\,{\text{br}}.)_{i} + {\mathbf{z}}^{\prime}_{i} {{\varvec{\upbeta}}}_{4} $$

Blanchard’s equations may be somewhat confusing, in that the variable Older Siblings estimates the effect of older brothers in the equation containing the variable Older Sisters, whereas Older Siblings estimates the effect of older sisters in the equation containing the variable Older Brothers (see Blanchard, 2022, Table 1). Therefore, Zdaniuk et al. (2025) further rearranged the predictor variables in Blanchard’s two equations in a way that produces the same quantitative output but makes the procedure easier to comprehend. Specifically, the first equation jointly considers numbers of siblings, older sisters, and older brothers, whereas the second equation is left unchanged:

5a $$ {\mathbf{x}}^{\prime}_{i} {{\varvec{\upbeta}}} = \beta_{0} + \beta_{1} N({\text{sib}}.)_{i} + \beta_{2} N({\text{older}}\,{\text{br}}.)_{i} + \beta_{3} N({\text{older}}\,{\text{sis}}.)_{i} + {\mathbf{z}}^{\prime}_{i} {{\varvec{\upbeta}}}_{4} $$

5b $$ {\mathbf{x}}^{\prime}_{i} {{\varvec{\upbeta}}} = \beta_{0} + \beta_{1} N({\text{sib}}{.})_{i} + \beta_{2} N({\text{older}}\,{\text{sib}}.)_{i} + \beta_{3} N({\text{older}}\,{\text{br}}.)_{i} + {\mathbf{z}}^{\prime}_{i} {{\varvec{\upbeta}}}_{4} $$

**Table 1:** Regression approach introduced by Ablaza et al. (2022), modified by Blanchard (2022), and reorganized by Zdaniuk et al. (2025)

Predictor variable	Interpretation of regression coefficient	Full label	Acronym
*Regression Eq. *1			
Number of siblings	One younger sibling is added to sibship	Younger sibling addition effect	YSAE
Number of older brothers	One older brother replaces one younger sibling	Older brother swap effect	OBSE
Number of older sisters	One older sister replaces one younger sibling	Older sister swap effect	OSSE
*Regression Eq. *2			
*Number of siblings*	*One younger sibling is added to sibship*	*Younger sibling addition effect*	*YSAE*
*Number of older siblings*	*One older sister replaces one younger sibling*	*Older sister swap effect*	*OSSE*
Number of older brothers	One older brother replaces one older sister	Brother–sister swap effect	BSSE

Redundant parameters are printed in italics

Because of its somewhat greater transparency, the Zdaniuk et al. version is the one that we will present in this article. We will refer to this as the ABZ (*A*blaza et al., Blanchard, and *Z*daniuk et al.) model.

## Using the ABZ Model

Table 1 summarizes the organization and interpretation of the key predictor variables listed in Eq. 5a and 5b. The predictor variables are shown in the first (i.e., leftmost) column of the table. Each predictor variable generates a different regression coefficient, and our interpretation of that regression coefficient is shown in the second column. This column, “Interpretation of regression coefficient,” is intended to explain the result for each variable as if it had been produced by means of experimental design rather than multiple regression. Take, for example, the interpretation for the variable Number of Older Sisters in regression Eq. 1 (“One older sister replaces one younger sibling”). The observed regression coefficient is analogous to the result that would be obtained by comparing the prevalence of homosexuality in two groups, the first group having one more older sister than the second group and the second group having one more younger sibling (the reason for the hypothetical comparison group having one more younger sibling is to keep total sibship size the same).

The third column, “Full label,” presents the name we have given to each parameter estimate (i.e., regression coefficient). These names basically summarize the interpretation of each parameter estimate. The initialisms given in the fourth column are simply short forms of the longer full labels. Thus—continuing the previous example—Older Sister Swap Effect (abbreviated OSSE) refers to the change in the odds of homosexuality when one older sister replaces one younger sibling in an otherwise unaltered sibship.

As we explained earlier in this paper, we prefer to use labels related to our operational definitions of sibship parameters when we are talking about observed results, as opposed to theoretical entities. Thus, YSAE represents the sibship size effect, OBSE represents the fraternal birth order effect, OSSE represents the sororal birth order effect, and BSSE represents the difference in magnitude between the fraternal and sororal birth order effects.

The foregoing is straightforward, but other content in Table 1 might be puzzling. The same predictor variable, number of Older Brothers, estimates two different things in the two equations. In Eq. 1, it estimates the effect of older brothers on the odds of homosexuality. In Eq. 2, it estimates the difference between the effect of older brothers and the effect of older sisters. This is possible because the “meaning” of a regression coefficient associated with a given predictor changes according to the other predictors in the equation. This is, of course, generally true in multiple regression. In the Ablaza et al. method and its offshoots, this fact is leveraged to produce coefficients with specific, desired interpretations. In the interest of keeping the focus of this part of our article on the practicalities of running and interpreting an ABZ regression analysis, we will defer a more detailed explanation of this matter to a later section.

### Comparison with Ablaza et al. (2022)

The seminal article by Ablaza et al. (2022) includes tables similar to Table 1. Comparison of their tables with the present table will reveal one difference that has the potential to confuse readers. The present writers identify the OBSE with the fraternal birth order effect. In contrast, Ablaza et al. identified the BSSE with the fraternal birth order effect. Ablaza et al. did not even compute the OBSE, although they did discuss how this could be done.

Ablaza et al.’s decision to regard the BSSE as the most verisimilitudinous representation of the fraternal birth order effect can be understood in context. Prior to the time of their methodological work, the bulk of empirical research suggested that older sisters have no effect on the odds of homosexuality in later-born children, and this finding was also reflected in the theoretical mechanisms that were proposed to explain the fraternal birth order effect. Since the authors wanted to test for the existence of a fraternal birth order effect that was consistent with these theoretical underpinnings, they deemed it more appropriate to compare the effect of one older brother with the effect of one older sister. However, with the mounting evidence that older sisters might also have an effect on sexual orientation, this representation is becoming increasingly problematic. Therefore Blanchard (2022) identified the OBSE with the fraternal birth order effect, and this practice has been followed in subsequent studies (Fořt et al., 2024; Kabátek & Blanchard, 2025; Semenyna et al., 2023; Zdaniuk et al., 2025).

### A Worked-Out Example

It is often helpful to explain a statistical procedure with the aid of a worked-out example. To this end, we reanalyzed raw data from Blanchard et al. (1998). The subjects were 225 heterosexual and 385 homosexual male community volunteers. We carried out the ABZ regressions using SPSS Version 28. SPSS is a popular statistics program that is likely to be familiar to many readers. The results are shown in Table 2.

**Table 2:** Blanchard et al. (1998) data reanalyzed with ABZ model

Predictor variables	B	SE	Wald	df	Sig	Exp(B)
*Equation *1						
All Siblings (YSAE)	0.103	0.078	1.778	1	.182	1.109
Older Brothers (OBSE)	0.286	0.140	4.185	1	.041	1.331
Older Sisters (OSSE)	− 0.166	0.127	1.713	1	.191	0.847
*Equation *2						
*All Siblings*	*0.103*	*0.078*	*1.778*	*1*	*.182*	*1.109*
*Older Siblings*	*− 0.166*	*0.127*	*1.713*	*1*	*.191*	*0.847*
Older Brothers (BSSE)	0.452	0.181	6.225	1	.013	1.572

The column headed Exp(B) denotes the odds ratio, *e*^*B*^, that is, the antilog of the regression coefficient B. Redundant results are printed in italics

Table 2 presents both the regression coefficient (*B*) and the corresponding odds ratio (*e*^*B*^) for each predictor variable in each equation. The important results are shown in regular font; redundant results are shown in italics.

The first three important results come from the first equation. The results for the predictor variable All Siblings show that the corresponding regression coefficient was not significant, *B* = 0.103, *p* = .182. Thus, there was no discernable relation between sibship size and sexual orientation (YSAE). The results for the predictor Older Brothers show that there was a positive relation between number of older brothers and sexual orientation (OBSE), *B* = 0.286, *p* = .041. In contrast, the results for Older Sisters indicate no significant relation between number of older sisters and sexual orientation (OSSE), *B* = − 0.166, *p* = .191.

The fourth important result comes from the second equation. The results for the predictor variable Older Brothers show that the difference in magnitude between the effect of older brothers and the effect of older sisters (BSSE) was statistically significant, *B* = 0.452, *p* = .013. The odds ratio corresponding to this regression coefficient, *e*^0.452^ = 1.572, indicates that an individual with an older brother would have about 57% greater odds of homosexuality than a comparable individual with an older sister.

We indicated earlier in this article that the second regression equation was needed to generate a fourth parameter estimate. In the ABZ set-up, this fourth parameter is the BSSE. It is worth pointing out that the same parameter can be calculated from the results of Eq. 1 as OBSE – OSSE = 0.286 − (− 0.166) = 0.452. This operation does not, obviously, yield inferential statistics for the BSSE; however, these can be obtained by conducting a two-sided test of coefficient equality, with the null hypothesis being OBSE = OSSE. This computation, which is easily done in some statistical software packages, would thus eliminate the need to run Eq. 2 (Table 1).

## The Ceteris Paribus Condition and the Rationale of the Ablaza et al. (2022) Approach

We stated earlier that we would revisit the interpretation of parameter estimates in the Ablaza et al. method and its offshoots. We will do that in this section.

The plain-language interpretation of the regression coefficients in models based on Ablaza et al. derives from the combined presence of two separate conditions. The first of these is the familiar *ceteris paribus* condition that applies to multivariate regression models in general. The second is the special property of the utilized predictor variables, which is that some predictors are subsets of other predictors (for example, in Eq. 5a, the Numbers of Older Brothers and Older Sisters are both subsets of the superset predictor Number of Siblings).

The *ceteris paribus* condition maintains that each coefficient in the multivariate regression model can be interpreted as an estimator of the change in the outcome variable that is associated with a one-unit increase in the given predictor variable, other things held equal. For example, in the B&B regression model, the coefficient on the Number of Older Brothers quantifies the impact of a one-unit increase in the number of older brothers on the odds of homosexuality, while holding the other predictors (numbers of older sisters, younger brothers, and younger sisters) fixed. Because the numbers of other siblings are unchanged, the coefficient corresponds to the hypothetical scenario in which an older brother is added to an existing sibship.

The “subsetting” property of the predictors in the ABZ model makes the coefficient interpretations more elaborate. Consider the model in the first regression equation of Table 1. In this model, the coefficient on the Number of Older Brothers no longer represents the impact of adding one additional older brother to an existing sibship, because doing so would increase the Number of Siblings and thus violate the *ceteris paribus* condition. Instead, the coefficient in question represents the hypothetical scenario in which the number of older brothers increases by one while the numbers of siblings and older sisters are kept fixed.

In this scenario, to keep the same number of siblings, the increase in the number of older brothers must be compensated by a decrease in the number of other siblings within the sibship. We cannot decrease the number of older sisters, because the *ceteris paribus* condition mandates this category to be fixed as well. Therefore, the increase in the number of older brothers must be compensated by a decrease in the number of younger siblings (who comprise the omitted sibling category that does not need to remain fixed). The “swap” terminology outlined in Table 1 follows naturally from the compensatory nature of these hypothetical sibship exchanges.

## Comparison of Ablaza et al. (2022) and B&B Approaches

The Ablaza et al. regression approach has three advantages over the B&B regression approach. The first two advantages are features that the Ablaza et al. approach includes and the B&B approach simply lacks. First, it estimates the effect of family size, controlling for birth order. Second, it estimates the difference in magnitude between the effect of older brothers and the effect of older sisters.

The third advantage is not quite as simple as feature-present vs. feature-absent. Both approaches estimate the effects of older brothers and older sisters on the odds of homosexuality—that is their main raison d’être—but the Ablaza et al. approach explicitly controls for family size in these estimates.

It is useful to know how much and in what circumstances controlling for family size alters the results for older brothers and older sisters. That is because many previous studies have used the B&B regression model, and it is desirable to have some knowledge base for comparing them, at least roughly, with studies that use the Ablaza et al. model. The following section approaches this topic by comparing the ABZ and B&B models on purely mathematical–statistical grounds. As we will show, the two models will produce different results from the same data when the family sizes of the homosexual and heterosexual subjects are notably different from each other.

### Translation of Results Between Models

Since both models operate with similar sets of explanatory variables, it is possible to use the regression coefficients corresponding to the B&B model to approximate the regression coefficients corresponding to the ABZ method, if it were applied to the same data. This can be done by employing a few simple transformation equations. Consider the four B&B coefficients presented in Eq. 1, which we denote here *β*(Older Brothers), *β*(Older Sisters), *β*(Younger Brothers), and *β*(Younger Sisters). The approximate ABZ estimates can be obtained as follows:

6a $$ {\text{YSAE}} \approx \frac{{\beta \left( {\text{Younger Sisters}} \right) + \beta \left( {\text{Younger Brothers}} \right)}}{2} $$

6b $$ {\text{OBSE}} \approx \beta \left( {\text{Older Brothers}} \right) - \underbrace {{\frac{{\beta \left( {\text{Younger Sisters}} \right) + \beta \left( {\text{Younger Brothers}} \right)}}{2}}}_{{{\text{YSAE}}}} $$

6c $$ {\text{OSSE}} \approx \beta \left( {\text{Older Sisters}} \right) - \underbrace {{\frac{{\beta \left( {\text{Younger Sisters}} \right) + \beta \left( {\text{Younger Brothers}} \right)}}{2}}}_{{{\text{YSAE}}}} $$

6d $$ {\text{BSSE}} \approx \beta \left( {\text{Older Brothers}} \right) - \beta \left( {\text{Older Sisters}} \right) $$

The approximative nature of these transformation equations stems from the fact that the B&B model controls for the sexes of younger siblings, whereas the ABZ model (in its most parsimonious version) does not. Therefore, to obtain an approximate YSAE, we take the arithmetic mean of the B&B coefficients corresponding to adding a younger brother and a younger sister to the sibship (Eq. 6a). Equation 6b shows us that the (dis)similarity of OBSE and the B&B measure of fraternal birth order effect, $\beta ({\text{Older}}\;{\text{Brothers}})$, rests on the magnitude of YSAE. If YSAE is close to zero (meaning that family size per se wields no influence on sexual orientation), then the two measures will yield effects of similar magnitudes. However, if YSAE proves to be a strong predictor of sexual orientation (as in Ablaza et al., 2022), then the two measures will yield distinct effects at distinct levels of statistical confidence. In such a situation, $\beta ({\text{Older}}\;{\text{Brothers}})$ confounds the influence of older brothers with the influence of family size, whereas OBSE isolates the influence of older brothers, holding family size fixed. Equation 6b shows that the same reasoning applies to OSSE and $\beta ({\text{Older}}\;{\text{Sisters}})$.

Besides elucidating the confounding role of family size, Eqs. (6a)–(6d) are also valuable for acquiring an intuitive understanding of the relation between the ABZ and B&B methods. For example, they show that we can arrive at the OBSE by taking the effect of adding one older brother to a sibship (as computed by the B&B method) and combining it with the effect of removing one younger sibling from that sibship. In doing so, we are effectively operationalizing the logic of the OBSE thought experiment, as defined in Table 1: One older brother replaces one younger sibling. Thus, we can get the ABZ coefficients from B&B coefficients simply by looking at Table 1 and using the corresponding ABZ interpretations as blueprints for the mathematical operations that need to be applied to the B&B coefficients.

We can consider the BSSE as a second example. According to Table 1, the interpretation of the BSSE is that we replace one older sister with one older brother. This translates into taking the B&B effect of one older brother and subtracting the B&B effect of one older sister. We believe that seeing this mathematical correspondence will help practitioners to understand how the two methods (and their regression coefficients) relate to each other. This explanatory function of the transformation equations is separate from any other use they might have, e.g., compiling estimates for a meta-analysis.

Next, using data introduced earlier, we can explore the degree of agreement between the ABZ coefficients estimated directly by applying the ABZ model, and ABZ coefficients calculated indirectly by applying Eq. (6a)–(6d) to the coefficients estimated using the B&B model. Table 2 of the present article shows the four ABZ coefficients estimated directly from the raw data of Blanchard et al. (1998). The data needed to calculate these coefficients via the transformation equations are found in Table 2 of Blanchard et al. (1998), which reports the results of a B&B-type regression analysis.

The directly estimated YSAE is 0.103. The indirect YSAE is $\frac{{\left( {0.07 + 0.13} \right)}}{2} = 0.10$.

The directly estimated OBSE is 0.286. The indirect OBSE is $0.39 - 0.10 = 0.29$.

The directly estimated OSSE is − 0.166. The indirect OSSE is $- 0.06 - 0.10 = - 0.16$.

The directly estimated BSSE is 0.452. The indirect BSSE is $0.39 - ( - 0.06) = 0.45$.

The values in this example show a very close agreement. It must be recognized, however, that their similarity can be distorted if the sample contains very unequal numbers of younger brothers and younger sisters, or if these predictors exhibit unusual correlations with the rest of the predictors.

A complete explication of the relations between the B&B and ABZ models requires transformation equations for standard errors as well as for *β* coefficients. We show how such transformation equations are developed in Appendix. Transformation equations for standard errors may turn out to be useful in future statistical development. One might think they would already be useful in transforming B&B findings so that these could be combined with ABZ findings in meta-analyses. Unfortunately, these equations require covariance matrices from the original (i.e., B&B-type) study, and such matrices are virtually never reported. Thus, although meta-analyses that combine transformed results from B&B studies with results from ABZ studies are readily possible in theory, they would be difficult in practice.

## Application of the ABZ Model to Outcomes Besides Homosexuality

The ABZ regression approach, or its earlier versions (Ablaza et al., 2022, slightly modified by Blanchard, 2022), has recently been used to explore relations between sibship and minority sexual orientations other than homosexual sexual orientation as well as constructs related to sexual orientation such as handedness (Lalumière et al., 2000). In addition to proposing a modification to the Ablaza et al. method, Blanchard (2022) found that the likelihood of bisexuality in men increased with more older brothers and with more older sisters whereas it decreased in women with higher number of older sisters (reversed OSSE). Somewhat similar findings were reported by Zdaniuk et al. (2025) who, using the ABZ approach, found that having fewer older sisters was associated with a higher likelihood of being bisexual or asexual among women, and having more siblings overall was associated with increased likelihood of being bisexual or asexual among men. Kabátek and Blanchard (2025) studied sexual orientation derived from reported sexual behavior rather than participants’ self-identification and used the method of Ablaza et al. with Blanchard modification to examine bisexual and asexual sexual orientation in women and men and found no links with participants’ birth order. The only effect they found was a negative association of sibship size and asexuality in women and men. Bartlett et al. (2024) used the Ablaza et al. with Blanchard modification method when studying sibship effects on handedness and found a sibship size effect for handedness in men, and birth order effects on handedness in women and men.

Future accumulation of studies using comparable methodologies and the ABZ approach, which allows for estimation of unconfounded sibship effects, may help resolve discrepant results reported for asexual and bisexual populations so far. Additionally, the accumulation of precise and unconfounded findings is of vital importance for future theorizing about origins of minority sexual orientations, given that the underlying mechanisms linking those constructs with sibship composition have either not yet been articulated or remain highly speculative.

## Potential Limitations

One potential limitation of the ABZ model is that it presents practitioners with more statistical complexity than does the B&B model; in particular, it requires a grasp of the “subsetting” property of the predictor variables. This is well justified, as the ABZ model produces more reliable coefficient estimates by unambiguously separating older brother or sister effects (OBSE and OSSE) from the family size effect (YSAE). However, the requirement itself can discourage practitioners from adopting the new method, and it can also lead beginner practitioners to draw wrong inferences from their analyses. These issues were among the primary motivations for us to write the present manuscript and elucidate the method at hand.

Second, accounting for the family size effect inevitably comes at the cost of statistical power (Raymond et al., 2025). Due to the intrinsic correlation between the number of siblings and number of older brothers and sisters, the OBSE and OSSE estimates produced by the ABZ model can be expected to have larger standard errors than the $\beta ({\text{Older}}\;{\text{Brothers}})$ and $\beta ({\text{Older}}\;{\text{Sisters}})$ estimates produced by the B&B model. Once again, this limitation is more than justified, as it ensures that the OBSE and OSSE coefficient estimates are unconfounded and have unambiguous interpretations. Furthermore, as long as the family size wields an independent influence over people’s sexuality, the larger standard errors are also wholly appropriate, as they capture the true degree of statistical uncertainty that underlies the coefficient estimates.^1^

It should also be mentioned that the ABZ model does not automatically protect against distortions in the data produced by stopping rules. A *stopping rule* is a statement of the factors that couples consider in deciding whether or not to have more children. One example would be “Continue having children until you produce a girl.” The presence of stopping rules must be investigated separately using other statistical procedures, as in Blanchard and Lippa (2007). It should be clear that the problem of stopping rule distortion is not limited to the ABZ model or related approaches (e.g., Ablaza et al.). As far as we can see, that problem would affect any statistical approach to studying birth order.

We would also like to touch on the related issue of causality. It needs to be emphasized that all the established methods for studying the influence of sibship characteristics on sexual preferences are strictly associational in their nature. For this reason, we caution readers about making explicit causal claims (e.g., “having an additional younger sibling will cause a decrease in the odds of homosexuality among the older siblings”). These claims are particularly tenuous in the presence of omitted variables that may be influencing both the sibship characteristics and ascertained sexual preferences (e.g., more traditional families having more children and being less accepting of the non-heterosexual preferences of their offspring). The resulting omitted variable bias will likely distort the causal interpretation of the corresponding coefficient estimate (in this case, the YSAE), as well as all the other coefficients produced by the same regression model.

## Appendix

As already stated in the main body of this article, a complete explication of the relations between the B&B and ABZ models requires transformation equations for standard errors as well as for β coefficients. In this Appendix, we show that the calculation of standard errors is arithmetically straightforward. The real problem for future researchers who might want to use such equations to re-analyze published data is that the derivation of standard errors for the composite coefficients requires regression output that is almost never included in published manuscripts. That required output is the covariance matrix:

$$ {\text{cov}}(\beta ) = \left[ {\begin{array}{*{20}c} {{\text{var}}(\beta_{1} )} & {{\text{cov}}(\beta_{1} ,\beta_{2} )} \\ {{\text{cov}}(\beta_{1} ,\beta_{2} )} & {{\text{var}}(\beta_{2} )} \\ \end{array} } \right] $$

This matrix is part of the optional regression output produced by statistical software; in other words, researchers who run a regression using such software can request it to produce the covariance matrix along with the standard output. The diagonal terms can be obtained from published articles by squaring the standard errors for the respective coefficients; however, the off-diagonal terms are typically out of reach. Researchers who already have the covariance matrix, or who can obtain it from the original authors, can compute the standard errors of the composite coefficients (Eq. 6a–6d) by using the following equations:

$$ {\text{se}}(\beta_{{{\text{OBSE}}}} ) \approx \sqrt {\begin{array}{*{20}c} {var(\beta_{{N({\text{older}}\,{\text{br}}.)}} ) - cov(\beta_{{N({\text{older}}\,{\text{br}}.)}} ,\beta_{{N({\text{younger}}\,{\text{sis}}.)}} ) - cov(\beta_{{N({\text{older}}\,{\text{br}}.)}} ,\beta_{{N({\text{younger}}\,{\text{br}}.)}} )} \\ { + 1/2cov(\beta_{{N({\text{younger}}\,{\text{br}}.)}} ,\beta_{{N({\text{younger}}\,{\text{sis}}.)}} ) + 1/4{\text{var}} (\beta_{{N({\text{younger}}\,{\text{br}}.)}} ) + 1/4{\text{var}} (\beta_{{N({\text{younger}}\,{\text{sis}}.)}} )} \\ \end{array} } $$

$$  se(\beta _{{OSSE}} ) \approx \sqrt {\begin{array}{*{20}c}    {\text{var} (\beta _{{N(older\,sis.)}} ) - \text{cov} (\beta _{{N(older\,sis.)}} ,\beta _{{N(younger\,sis.)}} ) - \text{cov} (\beta _{{N(older\,sis.)}} ,\beta _{{N(younger\,br.)}} )}  \\    {\quad  + 1/2\text{cov} (\beta _{{N(younger\,br.)}} ,\beta _{{N(younger\,sis.)}} ) + 1/4\text{var} (\beta _{{N(younger\,br.)}} ) + 1/4\text{var} (\beta _{{N(younger\,sis.)}} )}  \\   \end{array} }      $$

$$ {\text{se}}(\beta_{{{\text{BSSE}}}} ) \approx \sqrt {var(\beta_{{N({\text{older}}\,{\text{br}}.)}} ) - 2cov(\beta_{{N({\text{older}}\,{\text{br}}.)}} ,\beta_{{N({\text{older}}\,{\text{sis}}.)}} ) + {\text{var}} (\beta_{{N({\text{older}}\,{\text{sis}}.)}} )} $$

$$ {\text{se}}(\beta_{{{\text{YSAE}}}} ) \approx \frac{{\sqrt {var(\beta_{{N({\text{younger}}\,{\text{sis}}.)}} ) - 2cov(\beta_{{N({\text{younger}}\,{\text{sis}}.)}} ,\beta_{{N({\text{younger}}\,{\text{br}}.)}} ) + {\text{var}} (\beta_{{N({\text{younger}}\,{\text{br}}.)}} )} }}{2} $$

## Footnotes

1 In the special case when family size can be safely deemed to wield no independent influence on sexual orientation, the B&B and ABZ methods will produce estimates of the same theoretical quantities (OBSE = $\beta (OlderBrothers))$, and the B&B method will yield a more precise set of estimates. However, establishing whether this special case applies in practice is challenging (it can only be done through imperfect heuristics), and so we advise the reader to err on the side of caution and default to the ABZ model instead.
